# Motion capture dataset of 137 post-operative total hip replacement patients performing activities of daily living

**DOI:** 10.1038/s41597-026-06925-w

**Published:** 2026-03-06

**Authors:** David E. Lunn, Enrico De Pieri, Graham J. Chapman, Morten E. Lund, Stephen J. Ferguson, Anthony C. Redmond

**Affiliations:** 1 Carnegie School of Sport, Leeds Beckett University, Leeds, UK; 2 NIHR Leeds Biomedical Research Centre, Leeds, UK; 3 Institute for Biomechanics, ETH Zurich, Zurich, Switzerland; 4 Allied Health Research Unit, University of Lancashire, Preston, UK; 5 AnyBody Technology, Aalborg, Denmark; 6 Institute for Rheumatic and Musculoskeletal Medicine, University of Leeds, Leeds, UK

**Keywords:** Skeletal muscle, Outcomes research, Muscle

## Abstract

Three-dimensional motion capture is a powerful tool in clinical and engineering applications, but it can be time consuming, expensive, and difficult to access patient populations. Here, we present an open-access dataset comprising 3D motion capture and ground reaction force data from 137 post-operative total hip replacement patients performing eight activities of daily living (ADLs): normal walking, fast walking, stair ascent, stair descent, sit-to-stand, stand-to-sit, lunge, and squat. Data were collected using a 10-camera Vicon system and two AMTI force plates, and are provided in C3D and txt file format, compatible with inverse dynamic analysis platforms (e.g Visual 3D, MOKKA) and musculoskeletal modelling platforms (e.g. OpenSim, AnyBody). Each file includes synchronised marker trajectories, analog force data, and participant metadata (e.g. age, sex, BMI, operated limb, time since surgery). This dataset offers a unique opportunity to study functional biomechanics across a range of ADLs, enabling applications in musculoskeletal modelling, rehabilitation assessment, and the development of machine learning algorithms.

## Background & Summary

Three-dimensional motion capture data has a wide range of applications including clinical evaluation of movement^1^, improving sports performance^2^ and medical device testing^3^. However, performing high quality marker-based motion capture analysis requires expertise, can be time-consuming, expensive, and sometimes it can be difficult to access patient populations. Datasets of 3D motion capture for walking^4^ and walking at various speeds^5^ are available and for most clinical questions and engineering solutions, where an understanding of human gait is required, self-selected walking speed is typically used. This is unsurprising, as it is the primary locomotive activity and is required for humans to be well-functioning. Walking is not, however, the only movement which is required for humans to be well-functioning and the ability to perform other ADLs is also relevant. These ADLs can be more demanding and add relevance to understanding wider patient function and well-being^6^. Previous research has shown that using a faster self-selected walking speed, and therefore eliciting a higher demand on the person, develops a greater understanding of a person’s functional capacity^7^. Furthermore, other more demanding activities, such as stair negotiating, standing from a seated position and lunging require larger joint excursions^8^, increased motor control^9^ and altered loading patterns. It is crucial, therefore, that researchers and clinicians better understand how patients function during these activities.

Here we present a unique dataset of 137 post-operative total hip replacement (THR) patients performing eight activities of daily living. These ADLs include, self-selected normal walking speed, self-selected fast walking, stair ascent, stair descent, squat, lunge, sit-to-stand and stand-to-sit. The selected ADLs, although not performed by everyone, are representative of activities commonly undertaken in daily life and previously referred to as activities of daily living^10–13^. Additional tasks, such as the lunge, were included to represent movements that increase joint excursion toward the end range of motion and replicate elements of sporting activities, including bowling and racket sports. The dataset is presented in C3D and txt file format, which can be used in most post-processing softwares for computational modelling e.g MOKKA, Visual3D, OpenSim, the Anybody Modeling System. The dataset includes data for marker trajectories and ground reaction force, and in addition, other meta information is included in the C3D files including patient age, sex, height, mass, operated limb and years since THR operation. A small subset of this dataset, containing data for three participants (participants codes- LLJ_029, LLJ_121 and LLJ_140), was previously made available as part of our publication^7^. This data is also available as part of this new dataset.

Whilst available datasets containing gait^4,14^ and other activities of daily living^15^ do exist, these typically focus on no more than two activities, healthy adults^16^, or heterogeneous populations^5^. For example, the dataset reported by Bertaux *et al*.^14^ provides rich biomechanical data collected pre- and post-total hip replacement (THR), offering valuable insight into early functional improvements following surgery. However, data collection in the Bertaux cohort was limited up to six months post-operatively, early in the recovery window and restricting interpretation of longer-term functional adaptation. In contrast, participants in our current dataset were assessed up to five years post-THR, enabling characterisation of longer-term functional changes that may not be captured during early recovery. Combining datasets such as these would allow complementary investigation of short- and long-term recovery trajectories following THR.

Similarly, existing datasets focusing on activities of daily living, such as Hanisch *et al*.^15^, include a limited range of tasks, primarily level walking and sit-to-stand transitions. While these activities are important for benchmarking functional performance in healthy populations and patient groups, they do not fully reflect the broader functional challenges encountered during everyday life. The current dataset extends beyond these tasks by incorporating a wider range of activities of daily living, providing greater ecological validity and improved insight into the functional demands patients must overcome to maintain an active and independent lifestyle.

The current dataset provides biomechanical data, and comprehensive demographic data, for multiple ADL’s performed by the same cohort of post-operative THR patients. This enables within-subject comparisons across tasks, offering unique insights into functional capacity, compensatory movement strategies, and inter-task variability in an older clinical population. This dataset has already been used to identify differences in THR patients when characteristics are stratified by age, gender, BMI and functional ability^7^, the dataset has provided an understanding of how joint contact forces change during different activities of daily living^8^ and how this has a subsequent impact on bone loading^17^. The dataset is now being released to allow wider use in musculoskeletal modelling, including potential contribution to artificial intelligence and machine learning driven models for training datasets.

## Methods

### Participants

137 post-operative total hip replacement patients (Female N = 70; age 71.5 ± 7.7 years; BMI 28.2 ± 3.9 kg/m^2^; years since operation 2.8 ± 1.4 years) were recruited into the study from a clinical database between September 2013 and October 2015. Inclusion criteria were total hip replacement between 1–5 years post-surgery, older than 18 years of age, no lower limb joint replaced other than hip joint(s), fully pain free and not suffering from any other orthopaedic or neurological problem which may compromise gait. Ethical approval was obtained via the UK national NHS ethics (IRAS) system and provided by the Health Research Authority Research Ethics Committee; Approval Number-14/NE/1013. All participants provided informed, written consent to take part in the study and for anonymised data to be used in future research by agreeing to the following statement “I give permission for the data collected in this study to be used in this and future related studies”.

The Microsoft Excel file titled Participant_Demeographics.xlsx (Supplementary Table 1) provides demographic and anthropometric data for all participants.

### Procedure

#### Experimental protocol

**Fig. 1:**
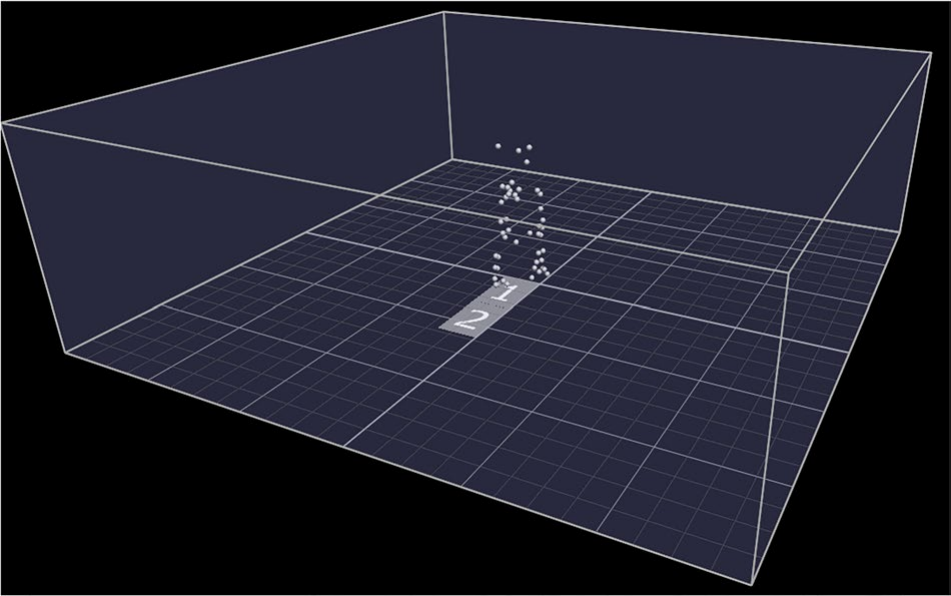
Schematic of the gait laboratory set up showing the capture volume, location of the two force plates and 3D tracking of the marker trajectories.

Lower limb kinematics and kinetics were collected using a ten camera Vicon system (Vicon MX, Oxford Metrics, UK) with a previously reported accuracy of 0.270 mm^18^, sampling at 100 Hz and integrated with two 600 mm × 400 mm force plates (AMTI, Watertown, MA, USA) positioned in the middle of the capture volume (6000 mm × 6000 mm × 2300 mm) capturing at 1000 Hz (Fig. 1). The Vicon motion capture system and AMTI force plates were synchronised through the MX Giganet, which acted as a central timing hub to ensure all data were collected on a shared timebase. The system was calibrated using an active wand and the laboratory coordinate centre (0,0,0) was located on the corner of the force plate in the same position for each patient using the calibration wand.

**Fig. 2:**
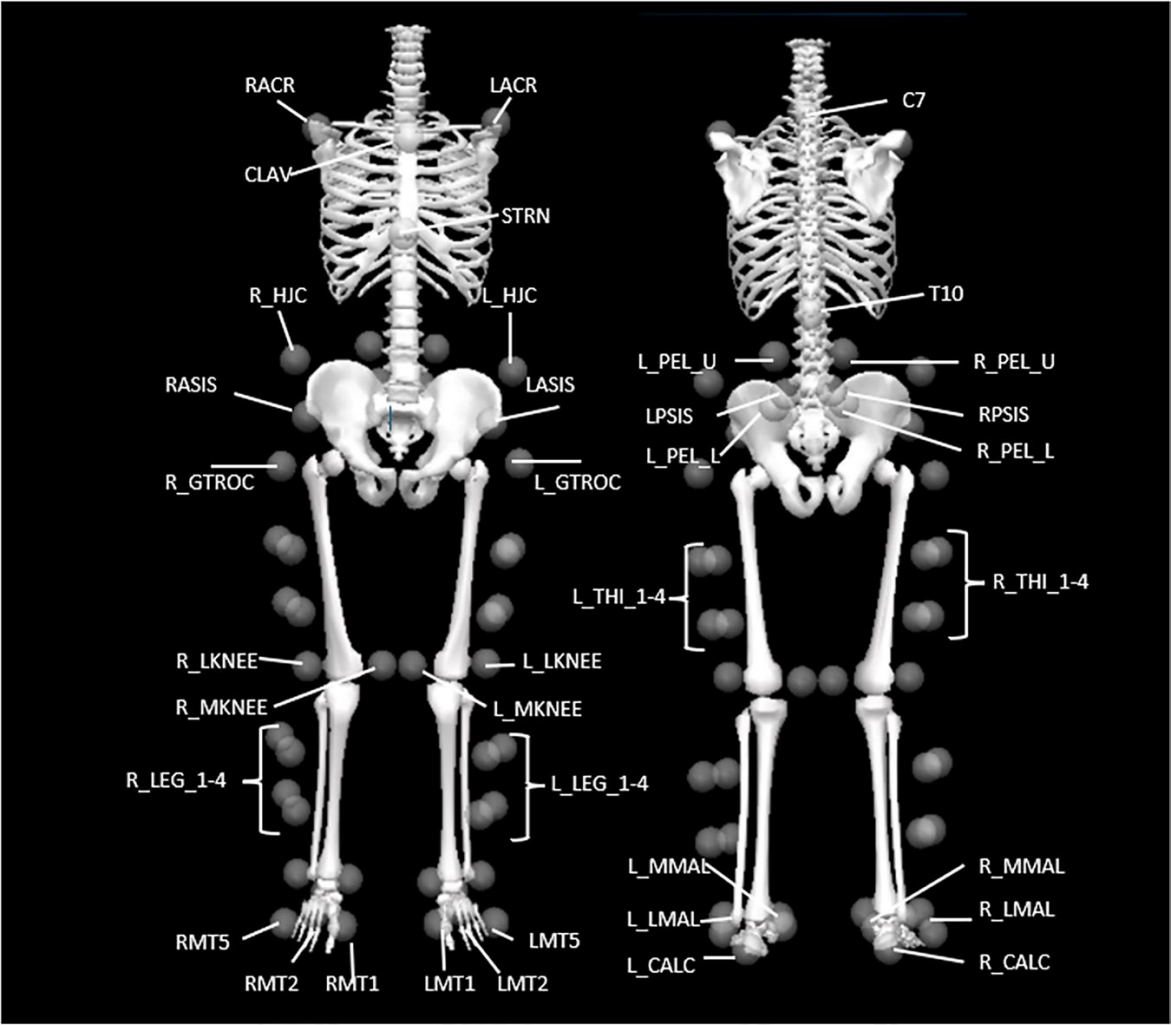
Anatomical locations of retroreflective markers displayed on reconstructed skeletal model. Anterior and posterior views are provided to facilitate visualisation of all marker positions. Markers were placed as defined by Van Sint Jan^36^. Table 1 provides a further description of these locations and marker names.

Retroreflective markers, for the lower body only, were placed in accordance with the calibrated anatomical system technique marker set to track lower limb segments kinematics in six degrees of freedom (three rotations about the X, Y and Z axes and three translations in the X, Y and Z directions), with four non-orthogonal marker clusters positioned over the lateral thighs, lateral shanks and sacrum^19^. With six retroreflective markers positioned on the first, second and fifth metatarsophalangeal joints as well as the malleoli and calcanei (Fig. 2 and Table 1). Participants wore tight fitting shorts and vest onto which reflective markers were affixed at bony anatomical landmarks using double-sided tape, to reliably determine anatomical joint centres. Before dynamic trials commenced, a static trial was collected to determine the position of the marker clusters with respect to lower limb joint location. Two additional markers were placed inferior to the malleolus markers during the stair negotiation trials to ensure tracking of the foot segment if the calcaneus marker was occluded by the stairs. Static trials were re-captured before the stair negotiation trials, due to these additional markers being applied.

**Table 1:** Description of the anatomical locations of the retroreflective markers.

Labels	Descriptions
ACR	Acromion Process
CLAV	Clavicle
STRN	Sternum
C7	7^th^ Cervical Spine
T10	10^th^ Thoracic Vertebrae
HJC	Iliac crest
ASIS	Anterior superior iliac spine
PSIS	Posterior superior iliac spine
GTROC	Greater Trochanter
PEL_U	Upper marker of pelvis cluster
PEL_L	Lower marker of pelvis cluster
THI_1–4	Thigh cluster markers
LKNEE	Lateral epicondyle
MKNEE	Medial epicondyle
LEG_1–4	Shank cluster markers
MMAL	Medial malleolus
LMAL	Lateral malleolus
CALC	Calcaneus
MT1,2,5	First, Second and Fifth Metatarsal head

#### Data processing

Labelling of the marker trajectories was performed in the Vicon Nexus software (Nexus 2.10, Vicon, UK). These trajectories were gap filled at a maximum of 10 frames using the Woltring spline algorithm (Woltring, 1986), following manufacturer recommendations (Vicon Nexus User Guide, v2.10). Data was then exported to Visual3D (HAS-Motion Inc., Kingston, ON) for further analysis and skeletal modelling. Marker coordinates data were filtered with a low pass Butterworth filter at 6 Hz cut-off frequency. Ground reaction forces and moments were smoothed using a 2nd-order lowpass Butterworth filter with a 25 Hz cut-off frequency. All forces and moments below a threshold of 5 N, defined on the vertical ground reaction force, were set to zero. The skeleton modelled in Visual3D, as reported elsewhere^7^, was used to define start and end events for all ADL’s. For all locomotor activities, gait events were identified using a pattern recognition based on kinematic data, with heel strike being identified using the vertical ground reaction force^20^. Details of the start and end event labels for the other ADLs are detailed below.

#### Experimental procedures

All data was collected during a single session, participants could choose not to complete the activities if they did not want to. Details of the number of activities completed by the participants is available in the Microsoft Excel file titled LLJ_Patient_Data.xls (Supplementary Table 2). All participants had a familiarisation period prior to completing 3–5 successful trials of each condition. Whilst all patients were screened during initial recruitment to ensure they could undertake the tasks required of them and were pain free, during the laboratory visit some patients could perform more trials than others and therefore the number of ADL’s and number of trials per ADL varied across the cohort. Each participant had a minimum of three trials and a maximum of six good trials per ADL which they undertook, details of these activities can be found in Participant Demographic.xlsx file. Individual trial recording duration was consistently set at ten seconds, allowing sufficient time for the movement to be captured. Files were then trimmed to the start and end of the ADLs. The activities of daily living are grouped into two categories: locomotor tasks (walk, fast walk, stair ascent, and stair descent) and non-locomotor tasks (sit-to-stand, stand-to-sit, squat, and lunge). All participants started testing with the walking trials followed by a fast walk and the sit-to-stand trials as these were the priority activities of the study, followed by the other ADLs which were randomised to try to minimise the effect of fatigue.

#### Walking (Number of trials (N)- 582; Average number of trials per patient (Avg)- 4);Fast walking (N-472; Avg- 4)

Participants undertook two walking conditions along a 10 m walkway (1) at a self-selected walking speed (hereafter referred to as a normal walk) and (2) a fast walk, where participants were instructed to walk “as fast as possible without running”. For analysis, only the gait cycles of successful trials were used. Where a successful trial was defined as a clean foot strike within the boundary of the force plate followed by a subsequent foot contact, of the same limb, within the capture volume. The gait events used in these trials were as follows; ON indicates contact with force place (R/LON), HS indicates heel strike or initial contact (R/LHS), OFF indicates leaving the force plate (R/LOFF) and TO indicates toe off the force or end of ground contact (R/LTO). The mean velocity for self-selected walking, derived from skeletal modelling, was 1.10 ± 0.17 m.s^−1^ (mean ± S.D) and 1.62 ± 0.24 m.s^−1^ for fast walking.

#### Stair negotiation- Ascent (N- 140; Avg- 3); Descent (N-136; Avg- 3)

Participants were asked to ascend and descend three steps at a self-selected speed, without the use of a handrail. The stairs were a cantilever design and bolted to the force plates^21^ to collect ground reaction force data on all three steps. The stairs measured 290 mm in tread depth, 170 mm in riser height, and 600 mm in width. Following the final step, a 1500 mm platform was provided to ensure the last step represented a true step and to allow participants to continue ambulating safely after stair ascent. Gait events were identified using the same method as described above. Heel strike (HS) refers to initial contact as some participants made contact with heel first and others with the forefoot first.

#### Standing and sitting transitions (N- 405; Avg- 3)

During the sitting and standing trials, participants sat on a platform with the feet shoulder-width apart, each foot positioned on a separate force plate in a fixed position. The chair was custom-built, with a cushioned seat attached to a hospital bed hoist, allowing adjustments to the seat height to standardise positioning between participants. The seat height was matched to the level of the patient’s tibial plateau, and the depth of the chair was 400 mm. Participants were then asked to stand, pause in their standing position for a brief moment, and return to a seated position without use of the arms which were held out straight ahead, to avoid any occlusion of the markers. The movement was split into a sit-to-stand and stand-to-sit phase. Initiation of movement (STS_Initiation) was defined by acromion marker velocity in the sagittal plane at a threshold of 0.1 m/s^−1^ and the stood-up position (STOOD_UP) was defined as the maximum coordinate value of the acromion marker in the transverse plane. Initiation of sitting (SITTING) was defined by the acromion marker falling below the maximum value and end of the sitting (END_SITTING).

#### Lunge (N-115; Avg- 3)

A lunge was chosen to replicate relevant sports activities such as lawn green bowls and tennis. Participants were asked to stand with both feet on one force plate and lunge forward, leading with the operated limb, onto the adjacent force plate then return to standing. The lunge action was split into two phases lunge descend and lunge ascend. Lunge initiation (LUNGE_INITIATION) was identified when the first metatarsal marker of the operated limb left the force plate and end of lunge descend (END_LUNGE_DESCEND) was defined when knee flexion was at the maximum value. This event also identified the start of the lunge ascend and the end of the lunge ascent was defined at the frame before the metatarsal made contact with the force plate (LUNGE_FINISH).

#### Squat (N-102; Avg- 3)

Squatting, or a variation of a squat, is performed on a daily basis as part of daily function^22^. Participants were positioned with one foot on each force plate, shoulder width apart, and were asked to perform a squat with arms out in front of them to avoid marker occlusion. The squat was split into two phases, squat descend and squat ascend. The start of the squat was defined by a change in the knee angular velocity (>10°/s)(SQUAT_INITIATION) and end of squat was defined at maximum knee flexion angle (BOTTOM_OF_SQUAT), and end of the squat was defined at maximum knee extension angle (END_SQUAT).

## Data Records

All data records are held in an open data repository managed by the University of Leeds and is available here 10.5518/1701^23^. This link contains all the data files and Microsoft Excel files which contains patient and trial information. A small subset of this dataset (comprising a limited number of participants and trials) was previously made available under 10.5518/319 as part of our previous publication^7^. The data records released previously were for three patients LLJ_029, LLJ_121 and LLJ_140. For clarity these files have been appended to version 2.0 in this release of the dataset and changed to version 1.0 in the previous release of data for these three patients. The current dataset represents the complete release, encompassing all participants and all recorded activities of daily living collected under the same methodological framework. The overlap is therefore limited to a few shared example files used for demonstration in the earlier publication. The two datasets are now explicitly cross-referenced to avoid confusion and to ensure transparency regarding data provenance.

### C3D files

Files are all stored in C3D file format (https://www.c3d.org). This file format is a public binary file format supported by all motion capture system manufacturers and biomechanics software programs.

### Files

The data is in a folder for each participant who took part in the study. The folder is labelled with an abbreviation of the study, LifeLongJoints (LLJ), and the participant ID number e.g LLJ_001, LLJ,002 etc. Each trial has at least one associated static trial, details of the trials for each patient and trial can be found in in the data repository stored as an excel spreadsheet (Trial_information.xlsx – Supplementary Table 2). These files detail which static trials are associated with which dynamic trial, there may be multiple static trials for each participant. If a retroreflective marker fell off during the dynamic trial another static was taken to ensure the markers accurately represented the anatomical landmarks. This file contains the following columns: Participant (e.g.LLJ_001), trial (e.g fast, walk, lunge), number (trial number), C3Dfile (name of the C3D file) and static (identifies which static trial is associated with the dynamic trial e.g. static_02). Dynamic trials were named according to the activity being recorded, followed by the trial number (e.g. walk_01, ascent_02). The following file naming conventions were used for each activity, as described in the experimental procedures: walk (walking), fast (fast walking), ascent (stair negotiation- ascending), descent (stair negotiation- descending), STS (sit-to-stand transitions), lunge (lunge), and squat (squat). Anthropometric and demographic parameters of each participant are stored in the excel file named “Participant_demographics.xlsx” (Supplementary table 2). This contains the study ID, date of collection, participant height (cm), mass (Kg), BMI, operated limb, age (years), sex, years since operation, and the number of trials in the dataset for each ADL. Whilst skeletal modelling in Visual3D was used to identify gait events, this information is not defined within the C3D files. As skeletal models can be software specific, only marker trajectories and force data were made available to ensure the dataset is more accessible.

### Data files

In addition to the C3D files, to make the data more accessible we have exported the data as txt files, which can be found in the participants individual folder. The variables contained in the txt files and their measurement units can be found in Table 2.

**Table 2:** Description of variables and units in.txt files.

Variable / Group	Description	Components	Units
**TARGET**	All target coordinates (C7, CLAV, L_CALC, etc.) representing anatomical landmarks and segments	X, Y, Z	Metres (m)
**EVENT_LABEL**	Event identifiers (e.g., Heel strike) more information can be found in the experimental procedures	—	Seconds (s)
**ANALOG.Fx1–Fz2**	Ground reaction force components from force plates 1 & 2	Fx, Fy, Fz	Newtons (N)
**ANALOG. Raw.pin 1–12**	The raw analog signal received from the force plates	Raw.Pin 1–12	Volts (v)
**ANALOG.Mx1–Mz2**	Moment components from force plates 1 & 2	Mx, My, Mz	Newton.metres (N·m)
**FORCE FP1,** **FP2**	Force plate identifiers	Fx, Fy, Fz	Newtons (N)

Kinematic target variables (TARGET) represent the three-dimensional Cartesian coordinates (X, Y, Z), measured in metres, of anatomical landmarks and segment reference points, and can be used to quantify segment position, displacement, and movement trajectories over time. Event labels identify discrete movement events (e.g. heel strike and toe-off) and are used to segment trials into start and end points. Further details of these can be found above in the experimental procedures.

Ground reaction force components (Fx, Fy, Fz) describe the external forces applied between the foot and ground in the medio-lateral, antero-posterior and vertical directions, respectively. Force plate moment components (Mx, My, Mz) represent the moments generated about the force plate axes. Force plate identifiers (FP1, FP2) indicate the source of force and moment data for each trial.

## Technical Validation

### Calibration of the optoelectronic system

The optoelectronic motion capture system (Vicon Motion Systems Ltd, Oxford, UK) was calibrated before each data collection session following the manufacturer’s recommended procedures. Calibration was performed using an active wand to establish the spatial relationships between cameras and define the global coordinate system. The active wand contained a series of infrared light-emitting diodes (LEDs) arranged at known fixed distances. During calibration, the wand was moved throughout the capture volume to optimise camera alignment and correct for optical distortion, enabling accurate 3D reconstruction through triangulation of the LED positions. The global coordinate system was subsequently defined using the wand, with the origin positioned at the laboratory floor located on the corner of the force plate for consistent positioning. A right-handed global laboratory coordinate system was defined with the Y-axis oriented antero-posteriorly, the X-axis medio-laterally, and the Z-axis vertically. Marker trajectory coordinates were expressed relative to this coordinate system. In all calibration files, residuals (i.e., the average of the 2D marker ray residuals corresponding to the same 3D point) were below 0.20 arbitrary units (Vicon), and the standard deviation of the reconstructed wand length remained below 1.5 mm (less than 1% of the wand length)

### Data validation

Whilst no formal validation has been conducted on the marker trajectories and force data released in this dataset, a robust calibration procedure, described above, was undertaken prior to every participant. Furthermore, data collection was undertaken in a Clinical Movement Analysis Society (CMAS) accredited laboratory and stringent standards for system calibration, data capture, and ongoing quality assurance were adhered to. Published work using this dataset has demonstrated that joint angles, joint moments, and joint contact forces fall within the expected ranges reported in previous studies using similar cohorts. For example our previous work^7^ reported hip sagittal plane range of motion of 31.03ᵒ to 36.71ᵒ; and hip frontal plane range of motion of 7.95ᵒ to 9.47ᵒ which are in the ranges of 30ᵒ−37ᵒ and 8ᵒ−10ᵒ, respectively, previously reported in the literature^24–29^. Furthermore, through musculoskeletal modelling, using this dataset peak hip joint contact forces during walking (~3–4 × body weight) and stair ascent (~4–5 × body weight) are comparable with those reported in a previous study measuring hip joint contact forces^30^. This demonstrates the validity of the current dataset for both kinematics and kinetics. However, due to the uniqueness of this dataset in this patient cohort some of the activities such as lunge, to our knowledge, do not exist therefore validation of these movements is difficult. Nevertheless, because data collection protocols were consistent across all ADL’s and the dataset aligns with established ranges for other ADLs, it is reasonable to expect that the same validity applies to the activities where no direct comparisons exist.

## Usage Notes

The recorded data are stored in C3D file format (https://www.c3d.org) and can easily be read using C3D toolboxes such as BTK (http://biomechanical-toolkit.github.io/) or EzC3D^21^. The software Mokka is a convenient tool for 3D visualisation (http://biomechanical-toolkit.github.io/mokka/index.html). It is commonly used to store, for a single trial, synchronised 3D markers coordinates and analog data (i.e. force plate recordings) as well as a set of metadata (e.g. measurement units, custom parameters specific to the manufacturer software application). As described in the data records, the data is also available in txt file format, permitting users to access and analyse the data through other platforms, such as MATLAB or Python.

The released motion-capture dataset, in C3D format, can also be used in conjunction with the musculoskeletal modelling software, AnyBody Modelling System (AnyBody Technology A/S, Aalborg, Denmark)^22^, allowing a potential user to carry out inverse kinematics and inverse dynamics analyses within the software. The models are made publicly available as part of the AnyBody Managed Model Repository (AMMR), thus guaranteeing compatibility with future releases of the software: https://github.com/AnyBody/Leeds-LifeLongJoints-Model. Whilst these model files contain no data associated with the dataset, the file structure is such that applying the C3D files to associated participant files would allow for straight forward analysis through the AnyBody software. As part of the research project LifeLongJoints personalised models for each participant were created from a detailed generic model of the lower limb^31^, based on a cadaveric dataset^32^, scaled to match the overall anthropometrics and the marker data collected during the static standing reference trial^33^. Muscle strength is scaled according to body mass and BMI of each participant, with the muscle fascicles modelled as constant strength actuators. Both implanted and native hip joints are modeled as a 3-degrees of freedom (DOF) ball-and-socket joint, while knee, talocrural, and subtalar joints are modeled as 1-DOF hinges and the position of the patella is defined as a function of the knee flexion angle^32^. Marker trajectories and GRF data from each motion-capture trial serve as input to trial-specific models, allowing to compute joint kinematics^34^, joint moments, joint reaction forces, as well as the muscle forces required to reproduce the given motion, through an inverse dynamics analysis based on a third-order-polynomial muscle recruitment criterion^35^.

## Supplementary information

Supplementary Table 1 - Participant Demographic Information

Supplementary Table 2 - Trial information

## Data Availability

The database is fully available at here 10.5518/1701^23^.

## Code availability

See usage notes for code availability for analysis.
